
JOURNAL INFORMATION
==============================
NLM Title Abbreviation: Buffalo Med J
NLM Journal ID: 0027534

Buffalo Medical Journal

ISSN: 1040-3817
Publisher: Biomedical Journal Digitization Project

ARTICLE INFORMATION
==============================
PMCID: PMC8735352
PMID: 36886824
Article version: 1
Subjects: Items

Items

Print publication date: 1905 Dec
Volume: 61
Issue: 5
First page: 334
Last page: 334
License: This work is free of known copyright restrictions. For more information, please see PMC Back Issue Digitization.
License URL: https://creativecommons.org/publicdomain/mark/1.0/

==============================

### ITEMS.

MESSRS. M. J. Breitenbach Company, 53 Warren Street, New York, are early in the field with their daily reminder, or Physician's Daily Memorandum, for 1906. It is one of the most useful belongings of the office desk with which we are familiar. We have used it for a number of years and should be sorry to have it discontinued. The proprietors of Pepto-Mangan (Gude) are entitled to receive, and we respectfully tender our best thanks for the continuation of this courtesy.

THE most popular dentifrice that has yet been introduced is Calox, the oxygen tooth powder. When brought into the presence of the saliva, oxygen is released which effectively destroys mouth bacteria and decomposing organic matter. Thus, it will be observed, it is a chemical agent as well as a mechanical cleanser. It may be confidently asserted that Calox is the only dentifrice that will sterilise the mouth and arrest caries without injury to the soft tissues. It is the most scientific tooth powder which the laboratory has yet produced.

THE Arlington Chemical Company, Yonkers, N. Y., continues to issue portfolios containing portraits of historic medical characters. The second in the series is a photogravure of Celsus. It is splendidly executed and is a valuable number of the collection. The fourth portrait is a fine picture of Galen which will appeal to every lover of classic medicine.

THE Pabst Extract Indian Calendar for 1906 is a decorative banner with an Indian's head in colors, and a monthly calendar on the obverse; on the reverse is an excerpt from Longfellow's song of Hiawatha, giving "Hiawatha's Wooing." It will be sent by the publishers, 930 Chestnut street, Milwaukee, on receipt of 10 cents.

---

WANTED.—Resident woman physician at Parkside Sanitarium, Amherst and Parkside Ave. Apply to Dr. Dunham, No 239 Delaware Avenue, Buffalo, N. Y.
